# *p*‐Orbital Ferromagnetism Arising from Unconventional O^−^ Ionic State in a New Semiconductor Sr_2_AlO_4_ with Insufficiently Bonded Oxygen

**DOI:** 10.1002/advs.202410977

**Published:** 2024-11-07

**Authors:** Xu‐Guang Zheng, Chao‐Nan Xu, Tomoki Uchiyama, Ichihiro Yamauchi, Tomasz Galica, Eiji Nishibori, Ying Chen

**Affiliations:** ^1^ Department of Materials Science and Engineering Faculty of Engineering Tohoku University Sendai 980‐8579 Japan; ^2^ Department of Physics Faculty of Science and Engineering Saga University Saga 840‐8502 Japan; ^3^ Department of Physics Faculty of Pure and Applied Sciences and Tsukuba Research Center for Energy Materials Science University of Tsukuba Ibaraki 305‐8571 Japan; ^4^ Fracture and Reliability Research Institute, Faculty of Engineering Tohoku University Sendai 980‐8579 Japan

**Keywords:** magnetic semiconductor, O^−^ ionic state, p‐orbital ferromagnetism, Sr_2_AlO_4_

## Abstract

Oxygen in solids usually exists in an O^2−^ ionic state. As a result, it loses its magnetic nature of a single atom, wherein two unpaired electrons exist in its outer 2*p* orbitals. Here, it is shown that an unconventional stable ionic state of O^−^ is realized in a new semiconductor material Sr_2_AlO_4_, leading to an intrinsic *p*‐orbital ferromagnetism stable until ≈900 K. Experimental and theoretical investigations have clarified that one‐fourth of the oxygen atoms in Sr_2_AlO_4_ are insufficiently bonded in the crystal structure, resulting in a unique O^−^‐state and *p*‐orbital ferromagnetism. To date, the O^−^ state is reported to exist only in non‐equilibrium conditions, and *p*‐orbital magnetism is only suggested in impurity bands with small ferromagnetic moments. The present work provides a new route for creating ferromagnetism in semiconductors and exploring new *p*‐orbital physics and chemistry. In addition, the material shows elastic‐mechanoluminescence that may enable unprecedented mechano‐photonic‐spintronics.

## Graphical abstract

An unconventional O^−^ ionic state and a high‐*T*_C_ *p*‐orbital ferromagnetism is discovered in a new semiconductor Sr_2_AlO_4_. The charge density contours show one less O─Sr bonding for the O4 site than the other sites of O1, O2, and O3. The insufficient bonding leads to the unconventional O^−^ ionic state and the unpaired *p*‐orbital electrons to result in stable ferromagnetism.

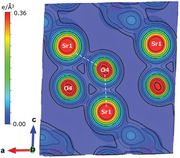

## 1 Introduction

One major type of solids is ionic solid, and the most important category is oxides, as exemplified in rechargeable batteries and high‐temperature superconductors. Oxygen is one of the most essential elements on earth, and the ionic state of oxygen in oxides profoundly affects the material's properties. The stable ionic state of oxygen in solids is considered to be O^2−^, but metastable states may exist under certain conditions. The best‐known example is in LiCoO_2_ for rechargeable lithium batteries^[^ ^1^ ^]^ and materials for future all‐solid‐state batteries, wherein an unusual oxygen ionic state has been reported to exist at non‐equilibrium. Another well‐known example is a possible deviation of oxygen ionic state in TiO_2_, etc., under UV radiation, leading to a photocatalyst effect to generate hydrogen from water.^[^ ^2^ ^]^ The anomalous high‐valence state is frequently discussed in transition‐metal oxides, such as in cuprate superconductors, wherein an ionic state of Cu more than Cu^2+^ is assumed. However, recent studies showed a slight deviation from the O^2−^ state for the oxygen ion instead of the ligand hole transferring from the metal ion to the O^2−^. Such states have been suggested or directly observed in various oxides such as CaCu_3_Fe_4_O_12_ and LaCu_3_Fe_4_O_12_,^[^ ^3^ ^]^ SrFeO_3_,^[^ ^4^ ^]^ NaCuO_2_,^[^ ^5^ ^]^ superconductors of YBa_2_Cu_3_O_7−𝛿_^[^ ^6^ ^]^ and La_3_Ni_2_O_7_.^[^ ^7^, ^8^ ^]^ It is widely believed that the ligand hole has an impact on the mechanism study of high‐temperature superconductivity.

A well‐known result of the O^2−^ ionic state in solids is losing the magnetic nature of a single atom, wherein two unpaired electrons exist in its outer 2*p* orbitals presented as ↑↓, ↑, ↑. However, here, we report a new semiconductor material of Sr_2_AlO_4_ that breaks this well‐accepted concept. Judging from the electron orbitals [Kr]5s^2^ in Sr and [Ne]3s^2^3p^1^ in Al, wherein the Sr^2+^ and Al^3+^ ionic states should be stable, an unconventional ionic state of oxygen can be expected. Indeed, we have verified this state and resulting *p*‐orbital ferromagnetism. Our experimental and theoretical investigations have clarified that one‐fourth of the oxygen atoms in Sr_2_AlO_4_ are insufficiently bonded in the crystal structure, resulting in a unique O^−^ state. This unconventional material shows an intrinsic *p*‐orbital ferromagnetism with an extraordinarily high Curie point. Up to date, there has been only suggestion that defects or impurities may produce spin‐split impurity bands near the fully occupied *p* orbitals (or *sp* hybrid orbitals),^[^ ^9^, ^10^ ^]^ which was proposed to account for the small ferromagnetic moment of dilute ferromagnetism in impurity‐doped or defected oxides.^[^ ^11^, ^12^, ^13^ ^]^ No intrinsic *p*‐orbital magnetism has been realized until the present work. The intrinsic *p*‐orbital magnetism in the present semiconducting material apparently has the advantages of stable ferromagnetism and relatively larger magnetic moment than the dilute or defected ferromagnetic semiconductors owing to the stable O^−^. It is of special interest to compare the present stable insufficient ionic bonding to the lasted finding of a stable insufficient covalent bonding that consists of carbon–carbon one‐electron *σ*‐bonding.^[^ ^14^ ^]^

## 2 Results and Discussion

### 2.1 The Unconventional Ionic State of O^−^

**Figure 1:**
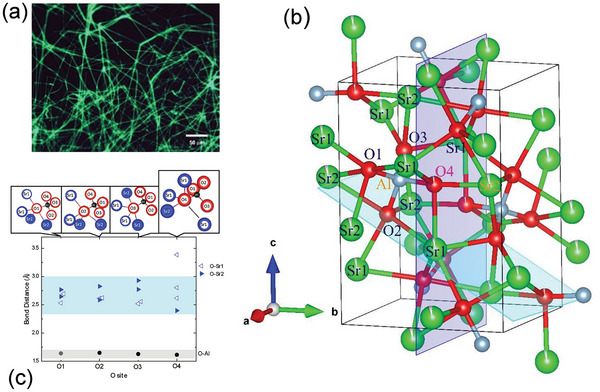
a) Whisker crystals of Sr_2_AlO_4_ under UV (*λ* = 365 nm) radiation showing luminescence. b) Crystal structure of monoclinic Sr_2_AlO_4_ at 30 °C, wherein the white thin slits inside Sr and O4 represent vacancy. Compared with O1, O2, and O3, O4 is insufficiently bonded with one less Sr atom. c) Bond lengths of O with surrounding Sr and Al atoms in Sr_2_AlO_4_ at 30 °C. Apparently, the O4 is insufficiently bonded with one less Sr (the next nearest Sr has a much longer distance of 3.3850(7) Å to O4 than other O–Sr atomic distances).

**Figure 2:**
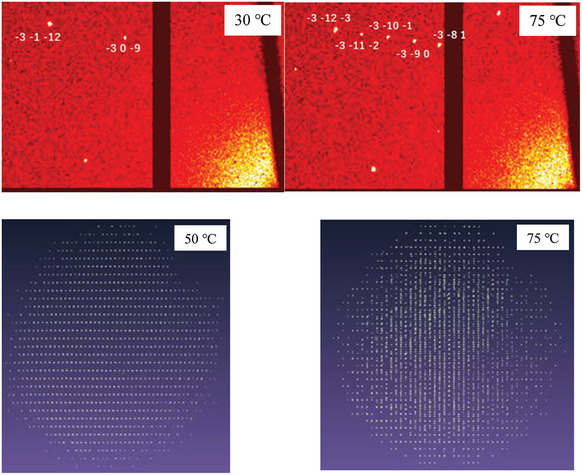
Synchrotron X‐ray diffraction patterns for single crystal Sr_2_AlO_4_. The experimental setup is displayed on the top with indexed diffraction spots at 30 and 75 °C demonstrating a structural transformation. The full diffraction patterns in the reciprocal lattice at 50 °C (viewed from the *b*‐axis) and 75 °C (viewed from the *a*‐axis) are exemplified corresponding to *b*×3 at 50 °C → *a* at 75 °C. Structures at 75 °C and above were twinned by inversion, i.e., with merohedral twin (‐100,0‐10,00‐1).

Synchrotron X‐ray diffraction of the whisker crystals (**Figure** **1**a) showed their single‐crystal nature, as demonstrated in **Figure** **2**. Under UV radiation, they showed luminescence which will be discussed later. A new structure Sr_2_AlO_4_ illustrated in Figure 1b (detailed structural Table S1, Supporting Information) has been revealed by structural analysis. As can be seen in Figure 1b and more clearly in Figure 1c, the most prominent feature of the structure is that the oxygen ion in the O4 site is insufficiently bonded with one Al and three Sr atoms (two Sr1 and one Sr2) while the O ions in other sites are all bonded with one Al and four Sr atoms (two Sr1 and two Sr2). In addition, a structural transformation from the monoclinic structure (Figure 1b) to the orthorhombic structure (Figure S2a, Supporting Information) occurred between 50 and 75 °C, which can be directly seen from the change in the diffraction spots in Figure 2, with an interchange of *a*→*c*, *b*×3→*a*, *c*→*b* (Table S2 and Figure S2b, Supporting Information). Of the twelve O sites in the high‐temperature phase, the O4, O8, and O12 are insufficiently bonded with one less Sr, which can be seen in the crystal structure in Figure S2a (Supporting Information) and in detail in Table S3 (Supporting Information). It should be noted that one‐fourth of the oxygen atoms in the orthorhombic structure are insufficiently bonded in a similar manner as in the monoclinic structure (as a matter of fact, the chemical bonding in these two structures is very similar because the monoclinic *β* = 92.635° is nearly vertical).

### 2.2 Ferromagnetism with High *T*_C_ ≈900 K

**Figure 3:**
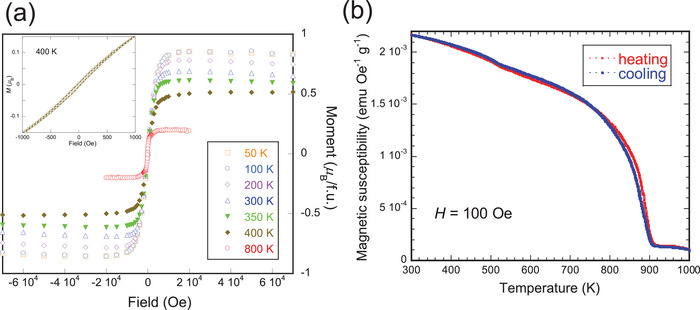
a) Magnetization versus field characteristics from 50 to 800 K for Sr_2_AlO_4_ with small hysteresis as illustrated in the inset plot. b) Magnetic susceptibilities measured at 100 Oe for Sr_2_AlO_4_ under heating and cooling conditions in a sweep mode at 1 K min^−1^. The small difference in the two curves seems to be caused by uneven thermal equilibrium.

One expected result of the unconventional O^−^ valence would be the unpaired electrons in its *p*‐orbital. Indeed, the new compound showed ferromagnetism with a saturated moment of ≈0.85 μB per formula Sr_2_AlO_4_ that justified this expectation (**Figure** **3**a). The *M–H* hysteresis is very small, which is thought to be a result of the small whisker diameters. The Curie point reached as high as *T*_C_ ≈900 K. The overlapping of magnetic susceptibilities upon heating and cooling suggested the stableness of the O^−^ state up to elevated temperatures (Figure 3b). Theoretical calculations predicted no change in magnetism upon structural transformation from the monoclinic to orthorhombic, which will be described later in this work.

### 2.3 Experimentally Observed Electronic Properties

**Figure 4:**
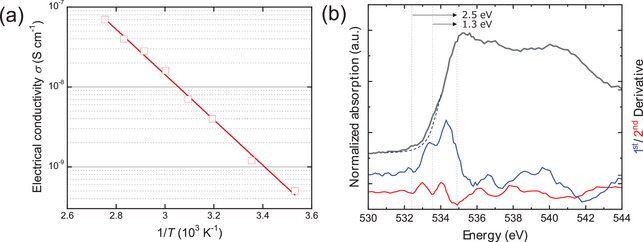
a) Electrical conductivity of a single whisker of Sr_2_AlO_4_ along the [110] crystallographic direction, showing the semiconducting property with energy gap ε_*g*_ ≈1.13(±0.25) eV. b) Oxygen 1*s* X‐ray‐absorption edges in Sr_2_AlO_4_ at 300 K, exhibiting two additional pre‐absorptions centered at *E* = 532.4 and 533.6 eV before the main absorption peak at 534.9 eV. The dashed line indicates an ordinary absorption curve that helps to see the pre‐absorptions. The complicated changes above 536 eV were due to multiple contributions from Al and Sr.

The Sr_2_AlO_4_ is semiconductive as presented in **Figure** **4**a. The estimated value of the energy gap *ε*_g_ ≈1.13(±0.25) eV corresponds to an intrinsic energy gap, as will be seen later. The nature of partially filled oxygen *p* orbitals has been experimentally verified by a synchrotron X‐ray absorption spectroscopy (XAS) study carried out in the bulk‐sensitive fluorescence mode. The XAS for O K‐edge absorption, which measured the absorption from the 1*s* orbital of oxygen to unoccupied orbitals, exhibited two additional pre‐absorption peaks at *Ε *= 532.4 and 533.6 eV, which are lower than the main absorption peak at 534.9 eV by 2.5 and 1.3 eV, respectively (Figure 4b). These two additional pre‐absorptions demonstrated that there were unusual unoccupied states of oxygen in the present crystal.

### 2.4 Theoretically Calculated Valence State and Electronic Properties

#### 2.4.1 The Unconventional Ionic State of O^−^

**Figure 5:**
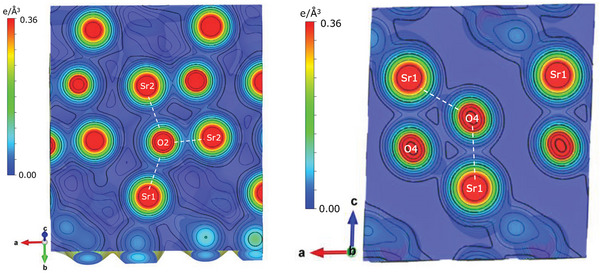
The projections of charge density contours (calculated) in the respective crystal planes are color‐painted in Figure b, showing the main configurational features of O2 and O4 atoms. The number of bonded Sr for O2 and O4 is three and two, respectively, in these planes, while there are one Sr and one Al for both O2 and O4 in the direction nearly vertical to the respective planes.

Density functional theory provides a powerful prediction and verification for crystallographic and electronic structures. The insufficiently bonded property of the O4, as illustrated in Figure 1, was further proven by the charge density distribution calculated using the DFT+*U* method (*U* = 12.0), which was optimized by reproduction of the crystal structure, and the experimentally observed electronic band structure as well. The charge density contours projected in the respective crystal planes color‐painted in Figure 1b apparently show one less bonding for O4 (**Figure** **5**). The ionic valences for all ions in the monoclinic structure were obtained from Bader analysis^[^ ^15^, ^16^ ^]^ to be +1.57, +1.53, +2.42, −1.54, −1.52, −1.49, and −0.97, respectively, for the Sr1, Sr2, Al, O1, O2, O3, and O4. The O4 has a low valence close to O^−^, which significantly deviates from the conventional O^2−^. This presents an interesting comparison to the slight valance deviation due to ligand hole transfer in other oxides and high‐temperature superconductors.^[^ ^3^, ^4^, ^5^, ^6^, ^7^, ^8^ ^]^

#### 2.4.2 The Electronic Properties

**Figure 6:**
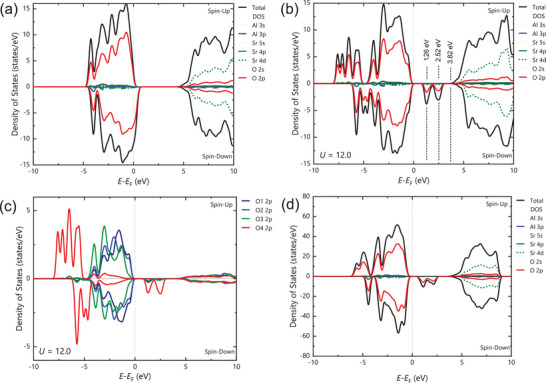
a) Density of states (DOS) calculated with the standard DFT (*U* = 0) for nominal composition Sr_2_AlO_4_ in monoclinic crystal structure. b) DOS for monoclinic structure Sr_2_AlO_4_ optimized with *U* = 12.0 eV in the GGA+*U* method. c) Decomposition of DOS of the O‐2*p* orbitals to the local DOS of oxygen atoms, showing that the uncancelled spins and the unoccupied O‐2*p* bands mainly arise from the O4 site. d) DOS for Sr_2_AlO_4_ in the orthorhombic structure.

The standard DFT calculation for nominal‐composition Sr_2_AlO_4_ in the monoclinic structure predicted asymmetric density of states (DOS) for spin‐up and spin‐down electrons below *E*_F_ that leads to a ferromagnetic moment of ≈1*μ*_B_/Sr_2_AlO_4_ (**Figure** **6**a), which accounts well for the observed ferromagnetism. However, the metallic band feature contradicted the experimentally observed semiconductive property and, moreover, the two additional pre‐absorptions in Figure 4. Since the preliminary work of Anisimov and co‐workers,^[^ ^17^ ^]^ it has been exemplified by numerous studies on various materials, especially transition‐metal oxides, that a Hubbard *U* correction (DFT+*U*) with the on‐site Coulomb interactions must be used to overcome limitations of standard DFT for strongly correlated systems.^[^ ^18^, ^19^, ^20^ ^]^ Our calculation using the DFT+*U* method suggests that the *U* = 12.0 eV produced lattice parameters nearest to the experimentally observed values in the monoclinic structure. The total energy of the spin‐polarized and non‐spin‐polarized calculations were −12.39 and −11.51 eV per molecule, respectively, leading to their formation energies of −2.289 and −2.237 eV per atom, respectively. Therefore, the ferromagnetic state is favored over the nonmagnetic state. The DOS calculated with this condition consistently matched the experimentally observed electronic properties, as shown in Figure 6b. The same ferromagnetic moment of ≈1*μ*_B_/Sr_2_AlO_4_ was obtained with the optimal *U* = 12.0 eV and the standard *U* = 0 despite their different level of *E*_F_. In particular, there are two minor unoccupied bands centered at *Ε* *− Ε*_F_ = 1.26 and 2.52 eV, which are lower than the main unoccupied band at *E−E*_F_ > 3.82 eV by 2.56 and 1.30 eV, respectively. They are in good consistency with the XAS pre‐absorption peaks in Figure 4b, whose differences from the main absorption peaks were 2.5 and 1.3 eV, respectively. Meanwhile, the calculated energy gap of 1.26 eV is consistent with the measured energy gap *ε*_g_ = 1.13(±0.25) in Figure 4a, taking into consideration the experimental error. The DOS for partial wave orbitals has shown that most of the ferromagnetic moments, as well as these two unoccupied bands, came from the O‐2*p* orbitals with minor contributions from Sr‐4*d* electrons (Figure 6b). Further decomposition of the O‐2*p* orbitals into the local DOS of O1‐O4 atoms has demonstrated that the O4‐2*p* orbital electrons produced a dominant contribution both to the ferromagnetic moment and the unoccupied minor bands (Figure 6c), which is in good consistency with the insufficient bonding property of the O4 site in the crystal structure. The calculation for the orthorhombic structure with optimal *U* produces similar asymmetric DOS resulting in an unchanged ferromagnetic moment of ≈1*μ*_B_/Sr_2_AlO_4_ (Figure 6d). It should be noted that both the standard DFT and the DFT+*U* produced an almost equivalent ferromagnetic moment for both the monoclinic and orthorhombic structures. For the real material, an additional minor contribution might come from the defective or hybrid bands, as in the dilute magnetic semiconductors.^[^ ^9^, ^10^, ^11^, ^12^, ^13^ ^]^

It is worth noting that a non‐existing material of SrAlO_3_, which also has unbalanced ionic valences, has been theoretically predicted in the Materials Project (mp‐978862). Ferromagnetic ordering arising from oxygen has been reported to produce a comparable magnetic moment of 0.98 μ_B_ fu^−1^. (The bandgap was severely underestimated by the semi‐local DFT method, such as the *U* = 0 case in the present work). Therein, we can expect a similar O^−^ state as in the practical material Sr_2_AlO_4_.

Another interesting property of the new semiconductor material is its co‐existing property of mechanically excitable luminescence (ML) in the elastic deformation range, i.e., elastic‐ML, which was reported for SrAl_2_O_4_, etc.^[^ ^21^, ^22^, ^23^, ^24^ ^]^. The elastic‐ML from a single Sr_2_AlO_4_ whisker can be seen with the naked eye (Video S1, Supporting Information). Like the application of SrAl_2_O_4_, the ML property would enable visualization of stress distribution and make it useful for early risk detection of fatigue cracks in constructors of pipelines and bridges, etc.^[^ ^25^ ^]^

## 3 Conclusion

In summary, an unconventional ionic state of O^−^ has been realized in a new semiconductor material Sr_2_AlO_4_. One direct result is an intrinsic *p*‐orbital ferromagnetism stable at high temperatures. Up to date, the O^−^ state has been reported only in non‐equilibrium conditions, and there has been only a suggestion of impurity‐induced spin‐split bands in relation to the *p*‐orbital with very small ferromagnetic moments at relatively low temperatures. Therefore, the present work provides a new route for creating ferromagnetism in semiconductors. The concept of the *p*‐orbital ferromagnetism due to insufficiently bonded oxygen in semiconductors can be applied to material designing, thus opening a new route for creating and engineering spintronics. In addition, the co‐existing property of elastic‐mechanoluminescence may enable unprecedented mechano‐photonic‐spintronics. Orbital physics for *d*‐electrons with Coulomb interaction has been extensively studied to account for high‐temperature superconductivity and colossal magnetoresistance.^[^ ^26^ ^]^ Rich phenomena can be explored by extending orbital physics to *p*‐orbitals as well as examining new orbital chemistry. On the latter, it is of high interest to compare the present stable insufficient ionic bonding to the lasted finding of a stable insufficient covalent bonding in carbon–carbon one‐electron *σ*‐bonding^[^ ^14^ ^]^;both update the present knowledge on chemical bonding and provide new insight into material design. The present work suggests that future material design for the realization of stable O^−^ and the resulting *p*‐orbital ferromagnetism may be assisted by a structural investigation of insufficient bonding of oxygen, theoretical calculations of charge density contours, and Bader ionic valence analysis, besides the non‐equilibrium solid–vapor synthesis conditions inferred in the Experimental Section.

## Conflict of Interest

The authors declare no conflict of interest.

## Author Contributions

X.G.Z. and C.N.X. contributed equally to this work. C.N.X. carried out the material synthesis and characterization, while X.G.Z. conducted the magnetic measurements. I.Y. performed supplementary experiments. E.N. and T.G. determined the crystal structure. T.U. conducted the XAS experiment, and DFT calculations were performed by T.U., C.N.X., X.G.Z., and Y.C. The final manuscript was written by X.G.Z.

## Supporting information



Supporting Information

Supplemental Video 1

## Data Availability

The data that support the findings of this study are available in the supplementary material of this article.
