# Supplementary material for: p‐Orbital Ferromagnetism Arising from Unconventional O− Ionic State in a New Semiconductor Sr2AlO4 with Insufficiently Bonded Oxygen
Source: Adv Sci (Weinh). 2024 Nov 7;12(1):2410977. doi: 10.1002/advs.202410977 (PMC11714225; doi:10.1002/advs.202410977)
Supplement: Supplementary file 1 — Supporting Information [file ADVS-12-2410977-s002.docx]

Supporting Information

*p*-orbital ferromagnetism arising from unconventional O^−^ ionic state in a new semiconductor Sr_2_AlO_4_ with Insufficiently Bonded Oxygen

Xu-Guang Zheng^1,2,*^, Chao-Nan Xu^1,*^, Tomoki Uchiyama^1^, Ichihiro Yamauchi^2^, Tomasz Galica^3^, Eiji Nishibori^3^, Ying Chen^4^

**Contents:**

Experimental methods and three supplementary tables showing detailed information on the crystal structures and bonding environments for oxygen ions, a figure of element analysis and a figure showing crystal structure at 75 ℃ and change of lattice constants with temperature are provided as **Supporting Materials**. A Supplemental Video is also provided showing the elastic-ML, *i.e.*, light emitting upon a mechanical stress, from a single Sr_2_AlO_4_ whisker embodied in resin.

4. Experimental Section/Methods

*Materials synthesis*: Sr_2_AlO_4_ with a micro amount of Eu were serendipitously prepared by solid-state reaction of mixed and pressed pellets of fine powders of high-purity SrCO_3_, Al(OH)_3_, 0.2 at% Eu_2_O_3_, and 1 wt.% H_3_BO_3_ at 1450 ^º^C in Al_2_O_3_ crucible with lid for 4 hrs in Ar-5%H_2_ atmosphere in a high-purity Al_2_O_3_ tubed furnace followed by slow cooling. The H_3_BO_3_ served as a flux and evaporated at high temperatures. Whisker-like Sr_2_AlO_4_ crystals were grown inside the Al_2_O_3_ crucible from the pellet surface to the surrounding Al_2_O_3_ crucible lid. The addition of a micro quantity of Eu was initially intended as an emission center for mechanoluminescence, as we did for the different compound SrAl_2_O_4_.^[14, 15]^ However, our preparation of Sr_2_AlO_4_ without the addition of Eu failed up to date, making us suspect that the Eu_2_O_3_ might have a catalyst effect on crystal growth. Plate-like SrAl_2_O_4_ crystals can be also obtained and mixed with the Sr_2_AlO_4_ crystals by slightly different pressure of the atmosphere, which has been confirmed to be critical to the successful growth of the whisker-like crystals. The crystal growth inside the crucible should be a result depending on the relatively high vapor pressure of the Sr component in a situation suspected to be like a CVD process.

*Measurements*: Single-crystal synchrotron X-ray diffraction experiment was carried out at BL02B1 beamline of SPring-8 synchrotron facility at wavelength 0.2481 Å. Data reduction and absorption correction were performed with CrysAlisPro software.^[27]^ All crystal structures were solved and refined with software of SHELXT,^[28]^ SHELXL^[29]^ and Olex2.^[30]^ Magnetization measurements were performed on an assembly of ~ 4 mg whiskers using a commercial SQUID with a high-temperature oven. The samples were sealed into a small quartz glass tube in Ar atmosphere for the high-temperature measurement. The electrical conductivity of a whisker sample in length of 638 *μ*m, diameter of 5 *μ*m was performed using the two-probe method with DC current of 0.4 nA. O K-edge soft X-ray absorption spectroscopy (XAS) was carried out in the bulk-sensitive fluorescence mode in BL7U at Aichi Synchrotron Radiation Center, Aichi Science & Technology Foundation, Aichi, Japan. The samples were mounted on scratched indium sheets, and the spectra were collected under the vacuum (~10^-8^ Pa).

*Theoretical calculations*: The structural and electronic properties for nominal composition Sr_2_AlO_4_ were calculated by spin-polarized Density functional theory (DFT) calculations using the projector augmented wave (PAW) method^[31]^ as implemented in the Vienna Ab initio Simulation Package (VASP),^[32-34]^ with incorporated the on-site Coulomb self-interaction potentials (LDA+U/GGA+U)^[35]^ under the calculation conditions of K-mesh: 7 × 5 × 5, cutoff energy: 500 (eV) and convergence for energy: 1.0×10^-8^ (eV). The GGA+U method was found more suitable for predicting the electronic structures in the present material.

*Statistics*: The software VESTA was used to visualize the crystal structure. The program package VASP was used for theoretical calculations. The magnetic moment was calibrated with the molecular weight of the Sr_2_AlO_4_ in the sample.

**Table S1**. Structure information for Sr_2_AlO_4_ at 30 ℃. A small amount of Eu ~ 0.8 at% preferentially entered the Sr2 site. Meanwhile, a notable amount of 3.4 at% oxygen vacancy was present in the O4 site.

**Table S2.** Structure information for Sr_2_AlO_4_ at 75 ^o^C. A monoclinic to orthorhombic structural transition occurred with an interchange of *a*→*c*, *b*×3→*a*, *c*→*b*. Oxygen vacancy occurred at sites O4, O8 and O12.

**Table S3.** Bond length of O with surrounding Sr and Al in Sr_2_AlO_4_ at 75 ℃, showing the insufficient bonding nature of oxygen ions in sites O4, O8 and O12.

|  | O-Al | O-Sr |
| --- | --- | --- |
| O1 | 1.621(3) Å | 2.512(3) Å, 2.593(3) Å, 2.688(3) Å, 2.881(3) Å, 3.252(3) Å |
| O2 | 1.640(3) Å | 2.522(3) Å, 2.560(3) Å, 2.696(3) Å, 3.116(3) Å, 3.191(3) Å |
| O3 | 1.648(3) Å | 2.616(3) Å, 2.634(3) Å, 2.642(3) Å, 2.788(3) Å, 3.076(3) Å |
| O4 | 1.618(3) Å | 2.393(3) Å, 2.594(3) Å, 2.867(4) Å, 3.364(4) Å |
| O5 | 1.646(3) Å | 2.620(4) Å, 2.625(3) Å, 2.630(3) Å, 2.779(3) Å, 3.070(4) Å |
| O6 | 1.654(3) Å | 2.501(3) Å, 2.678(3) Å, 2.684(3) Å, 2.806(3) Å, 3.262(3) Å |
| O7 | 1.606(4) Å | 2.530(3) Å, 2.570(3) Å, 2.746(3) Å, 3.004(3) Å, 3.223(3) Å |
| O8 | 1.616(3) Å | 2.403(3) Å, 2.610(3) Å, 2.728(3) Å, 3.679(3) Å |
| O9 | 1.636(4) Å | 2.499(4) Å, 2.626(4) Å, 2.724(4) Å, 3.003(4) Å, 3.078(4) Å |
| O10 | 1.607(4) Å | 2.510(4) Å, 2.587(4) Å, 2.936(4) Å, 2.949(4) Å, 2.978(4) Å |
| O11 | 1.649(4) Å | 2.606(3) Å, 2.636(4) Å, 2.758(3) Å, 2.813(5) Å, 2.875(5) Å |
| O12 | 1.611(3) Å | 2.399(3) Å, 2.591(3) Å, 2.922(5) Å, 3.316(5) Å |

**Figure S1**. A figure of element analysis.

(a)

(b)

**Figure S2**. (a) High-temperature (HT) phase in orthorhombic structure at 75 ℃. (b) The change of unit cell parameters showing a structural transition from monoclinic to orthorhombic between 50 ℃ and 75 ℃ with an interchange of a→c, b×3→a, c→b. The unit cell parameters are unified by the corresponding values in the room-temperature (RT) phase at 30 ℃.
